# Occulomotor Neural Integrator Dysfunction in Multiple Sclerosis: Insights From Neuroimaging

**DOI:** 10.3389/fneur.2018.00691

**Published:** 2018-08-23

**Authors:** Peter Bede, Eoin Finegan, Rangariroyashe H. Chipika, Stacey Li Hi Shing, Jeffrey Lambe, James Meaney, Janice Redmond

**Affiliations:** ^1^Computational Neuroimaging Group, Academic Unit of Neurology, Trinity College Dublin, Dublin, Ireland; ^2^Laboratoire d'Imagerie Biomédicale, Sorbonne University, CNRS, INSERM, Paris, France; ^3^Department of Neurology, St James's Hospital, Dublin, Ireland; ^4^Centre for Advanced Medical Imaging (CAMI), St James's Hospital, Dublin, Ireland; ^5^School of Medicine, Trinity College Dublin, Dublin, Ireland

**Keywords:** multiple sclerosis, nystagmus, brainstem, cerebellum, striatum, thalamus, hippocampus

## Abstract

**Background:** Magnetic resonance imaging is a key diagnostic and monitoring tool in multiple Sclerosis (MS). While the substrates of motor and neuropsychological symptoms in MS have been extensively investigated, nystagmus-associated imaging signatures are relatively under studied. Accordingly, the objective of this study is the comprehensive characterisation of cortical, subcortical, and brainstem involvement in a cohort of MS patients with gaze-evoked nystagmus.

**Methods:** Patients were recruited from a specialist MS clinic and underwent multimodal neuroimaging including high-resolution structural and diffusion tensor data acquisitions. Morphometric analyses were carried out to evaluate patterns of cortical, subcortical, brainstem, and cerebellar gray matter pathology. Volumetric analyses were also performed to further characterize subcortical gray matter degeneration. White matter integrity was evaluated using axial-, mean-, and radial diffusivity as well as fractional anisotropy.

**Results:** Whole-brain morphometry highlighted considerable brainstem and cerebellar gray matter atrophy, and the tract-wise evaluation of white matter metrics revealed widespread pathology in frontotemporal and parietal regions. Nystagmus-associated gray matter degeneration was identified in medial cerebellar, posterior medullar, central pontine, and superior collicular regions. Volume reductions were identified in the putamen, thalamus and hippocampus.

**Conclusions:** Multiple sclerosis is associated with widespread gray matter pathology which is not limited to cortical regions but involves striatal, thalamic, cerebellar, and hippocampal foci. The imaging signature of gaze-evoked nystagmus in MS confirms the degeneration of key structures of the neural integrator network.

## Introduction

Magnetic resonance (MR) imaging plays a central role in the diagnosis and monitoring of multiple sclerosis (MS) and is a key outcome measure of phase III clinical trials. While clinical radiology continues to focus on the estimation of white matter (WM) lesion load, recent imaging studies have been increasingly successful in capturing progressive cortical gray matter (GM) degeneration (1). Cortical pathology in MS has been evaluated by a variety of imaging techniques, including quantitative susceptibility mapping, (2) perfusion MR, (3, 4) magnetisation transfer ratio imaging, (5) proton spectroscopy (6) and functional network analyses (7). The drawback of these techniques is that they require carefully optimized sequences, time consuming data acquisitions and complex post-processing pipelines which make them better suited for research purposes than everyday clinical applications. While subcortical gray matter degeneration in MS has been associated with a number of non-motor symptoms, such as fatigue, (8–10) memory impairment, (11, 12) and depression, (13) these structures are difficult to evaluate qualitatively in a clinical setting. Cerebellar imaging in MS has also overwhelmingly focused on white matter metrics, even though cerebellar gray matter degeneration have been linked to motor disability, (14) impaired information processing, (15), and falls (16).

The pathological substrate of non-motor features of MS, such as pseudobulbar affect, fatigue, apathy, eye-movement disturbances, and neuropsychological deficits are relatively poorly characterized in contrast to the plethora of imaging studies focusing on motor disability and gait impairment. It is widely recognized that white matter pathology alone does not account for the heterogeneity of clinical manifestations in MS and that cortical and subcortical gray matter pathology contribute substantially to extra-motor deficits (17). While gaze-evoked nystagmus (GEN) is a common manifestation of MS, it is seldom evaluated specifically in dedicated imaging studies (18, 19). Nystagmus has considerable quality of life implications, it impacts on rehabilitation efforts, ability to work, reading, using computers, watching television, driving, and operating machinery (20). Additionally, nystagmus in MS has been linked to headaches, difficulties with concentration, and distractibility. GEN is widely regarded as a specific manifestation of neuronal integrator dysfunction, which provides insufficient discharge to fixate at an eccentric position, resulting in a drift to the primary gaze position and triggering a corrective saccade (21). Traditionally, an upper, middle and lower saccade pathway is distinguished, where the upper pathway contributes to saccade initiation and target setting (22, 23). The middle saccade pathway originates from the superior colliculus (SC) and innervates the lower saccade pathway which triggers the excitatory burst neurons (EBNs) in the paramedian pontine reticular formation (PPRF). The horizontal neural integrator is primarily located in the nucleus prepositus hypoglossi (NPH), and to a lesser extent in the medial vestibular nucleus (MVN) (22, 24). Pharmacological treatment options for GEN are limited (25). Baclofen is thought to increase the action potentials of Purkinje cells, thus enhancing neural step integrator firing (26, 27). Other drugs, such as Clonazepam, Gabapentin and memantine are often tried with varying efficacy (28, 29). Despite its high incidence, limited therapeutic options and quality of life implications, GEN in MS is surprisingly under investigated in MRI studies. Accordingly, we have undertaken a prospective imaging study of a cohort of MS patients who all had horizontal gaze-evoked nystagmus to assess cerebellar, brainstem, and subcortical gray matter degeneration in addition to white matter alterations.

## Methods

### Participants

A prospective multimodal neuroimaging study was undertaken with the participation of 31 multiple sclerosis patients and 20 age-matched healthy controls. Patients with multiple sclerosis were recruited from a specialist MS clinic in a tertiary referral center in St James's Hospital Dublin. Inclusion criteria included an established diagnosis of MS and persistent horizontal gaze-evoked nystagmus on clinical assessment. Patients were only included if they had had horizontal gaze-evoked nystagmus (GEN), (30) sustained, large amplitude, non-pendular, jerk nystagmus while attempting to maintain an eccentric eye position (30, 31). GEN and is easily distinguishable from physiological end-point nystagmus (EPN) and to ensure assessment uniformity all patients were assessed by the same experienced neurologist. Exclusion criteria included internuclear ophthalmoplegia, intracranial pathology other than MS, previous cerebrovascular events, prior neurosurgical intervention, uncontrolled hypertension, in addition to standard MR safety exclusion criteria. Based on the above criteria one healthy control was excluded due to an incidental intracranial finding; a meningioma, and one control's T1-weighted structural data could not be included due to movement artifacts. Nine MS patients have been excluded from diffusion tensor analyses due to poor quality raw data. All participants provided informed written consent in accordance with the ethics (IRB) approval of this study. The clinical and demographic profile of participants is summarized in Table 1.

**Table 1:** Demographic and clinical characteristic of patients and healthy controls.

**Neuroimaging data**	**3D structural T1-weighted data**			**32 Direction diffusion tensor imaging data**		
**Imaging analyses**	**Voxel based morphometry morphometry (cortical thickness) Subcortical gray matter volumes**			**Axial diffusivity (AD) Radial diffusivity (RD) Mean diffusivity (MD) Fractional anisotropy (FA)**		
**Cohort**	**Controls**	**MS**		**Controls**	**MS**	
n	18	31		19	22	
Mean age ± SD (y)	46.556 ± 12.6036	42.774 ± 8.6360	*p* = 0.219	47.211 ± 13.8547	43.955 ± 8.0621	*p* = 0.355
Gender, Male, n (%)	3 (16.7%)	8 (25.8 %)	χ^2^ = 0.148 *p* = 0.701	4 (21.1 %)	5 (22.7 %)	χ^2^ = 0.00 *p* = 1
Handedness, Right, n (%)	16 (88.88 %)	28 (90.32 %)	χ^2^ = 0.00 *p* = 1	17 (89.47 %)	20 (90.9 %)	χ^2^ = 0.00 *p* = 1
EDSS Median Range Interquartile range	N/a	3.5 1 – 6.5 2 - 6		N/a	4 1 – 6.5 2 - 6	
Bidirectional/Unidirectional nystagmus		18 / 13			13 / 9	
Mean disease duration ± Std. Deviation (y)	N/a	7.548 ± 4.2177		N/a	8.273 ± 4.1768	
Type of MS n (%)	N/a	RRMS 27 (87.1 %) SPMS 4 (12.9 %)		N/a	RRMS 20 (90.9 %) SPMS 2 (9.1 %)	

*PPMS, primary progressive MS; ROI, Region-of-interest; RRMS, relapsing-remitting MS; SPMS, secondary progressive MS*.

### MRI data acquisition

Magnetic resonance (MR) data were acquired on a 3 Tesla Philips Achieva system using an 8-channel receive-only head coil. T1-weighted images were acquired using a 3D volumetric fast gradient echo sequence with, spatial resolution = 1 × 1 × 1 mm^3^, field-of-view (FOV) of 240 × 240 × 163 mm, TR/TE = 25/2.1 ms, flip angle = 30°, SENSE factor = 2. Following file conversions and quality assessments, T1-weighted structural data were analyzed for 31 MS patients and 18 healthy controls. The parameters for fluid attenuated inversion recovery (FLAIR) imaging were the following: TR/TE, 11000/125 ms; TI: 2,800 ms; turbo factor: 31; refocusing angle; 120°; spatial resolution: 0.5 × 0.5 × 4 mm; SENSE: no. Diffusion tensor images (DTI) were acquired using a spin-echo planar imaging (SE-EPI) sequence with a 32-direction Stejskal-Tanner diffusion encoding scheme: FOV = 140 × 244 × 244 mm, 70 slices with no inter-slice gap, spatial resolution = 2 × 2 × 2 mm^3^, TR/TE = 12285/55 ms, SENSE factor = 2, *b*-values = 0, 1000 s/mm^2^, with SPIR fat suppression and dynamic stabilization in an acquisition time of 8 min 16 s. Following file conversions and quality assessments, diffusion tensor data was pre-processed for 22 MS patients and 19 healthy controls.

### Gray matter analyses

In order to reduce the effect of white matter (WM) lesions on tissue-type segmentation (32–34) and subsequent volumetric and morphometric analyses, lesion mapping and lesion filling was implemented on each participant's imaging data. White matter lesions were segmented by a single rater on FLAIR images and individual lesion masks were created for each participant using FMRIB's FSLeyes. Each subject's FLAIR image was co-registered onto their 3D T1-weighted structural image using FMRIB's Linear Image Registration Tool (FLIRT) (35, 36) and the resulting transformation matrix was then applied to the lesion map for registration onto the 3D T1-weighted image. The FSL lesion filling tool (34) was used to reduce intensity contrast within known lesion areas and thereby improve the accuracy of brain segmentation. This method uses the original T1-weighted structural image and the co-registered lesion mask to fill lesions with intensities matching the surrounding normal-appearing WM and has been shown to reduce tissue-type misclassification and improve the accuracy of subsequent brain volume measurements (34).

A dual approach was undertaken to comprehensively evaluate patterns of gray matter pathology; surface-based morphometry was carried out to identify focal cortical thinning and voxel-based morphometry (VBM) was performed to explore regional density alterations. For cortical thickness measurements, the FreeSurfer image analysis suite was used (37). The pre-processing pipeline included the removal of non-brain tissue, segmentation of the subcortical white matter and deep gray matter structures, intensity normalization, tessellation of the gray matter-white matter boundary, and automated topology correction (38). False Discovery Rate (FDR) corrections were used for group comparisons, statistical threshold set at *p* < 0.05 and contrasts were adjusted for both age and gender. The FMRIB's software library (FSL) (39) was used to perform VBM (40). Subsequent to brain extraction and tissue-type segmentation, gray-matter partial volume images were aligned to the MNI152 standard space using affine registration. The gray matter partial volume estimates were non-linearly co-registered to a study-specific template, modulated by a Jacobian field warp and smoothed with an isotropic Gaussian kernel with a sigma of 3 mm. The threshold-free cluster-enhancement (TFCE) method (41) and permutation-based nonparametric inference were used for the comparison of MS patients and healthy controls controlling for age and gender (42, 43). The statistical significance was set at *p* < 0.05 family-wise error (FWE). Total intracranial volume (TIV) was estimated for each participant by linearly aligning the subject's skull-stripped brain to the MNI152 space, computing the inverse of the determinant of the affine matrix and multiplying it by the size of the template. Following registration to template with FSL-FLIRT (35, 36), tissue type segmentation was undertaken using FSL-FAST (44).

### Subcortical gray matter analyses

In order to comprehensively evaluate subcortical gray matter degeneration, structures which exhibited density reductions on VBM were further evaluated by volumetric analyses using subcortical segmentation. Following standard pre-processing steps, the subcortical segmentation and registration tool FIRST (45) of the FMRIB's Software Library was used to estimate volumes of the thalamus, hippocampus, putamen, and brainstem. Pipelines for subcortical segmentation and volume estimations were previously described (46). Briefly, FSL-FIRST uses a two-stage affine registration algorithm to register input T1 data sets to the Montreal Neurological Institute 152 (MNI152) standard space and a model-based approach is then implemented for the segmentation of subcortical structures. Subcortical mesh and volumetric outputs are generated following automatic boundary corrections. Exploratory comparative statistics were carried out with IBM's SPSS Statistics version 22. Analyses of covariance (ANCOVA) were conducted to compare volumes of subcortical structures between study groups. Assumptions of normality, linearity and homogeneity of variances were verified. Volumes of subcortical gray matter structures were included as dependent variables, and study group allocation as the categorical independent variable. Age at the time of MRI scan, gender, and total intracranial volume (TIV) were used as covariates. Following Bonferroni corrections, a *p* < 0.0125 was considered significant. Effect-sizes were calculated using partial Eta squares (η2**)**. For illustrative purposes, boxplots of volumes were generated to highlight inter-group volumetric differences for each structure.

### White matter analyses

Total lesion load volume was estimated based on skull-striped FLAIR images using the pipeline described by Wetter et al. (47) in FMRIB's FSL environment. Pre-processing of raw diffusion tensor imaging (DTI) datasets included eddy current corrections, motion corrections and brain-tissue extraction using FSL (48). Subsequent to fitting a diffusion tensor model, maps of axial diffusivity (AD), radial diffusivity (RD) mean diffusivity (MD), and fractional anisotropy (FA) were generated. Tract-based spatial statistics (TBSS) and permutation-based nonparametric inference was used to compare the white matter profile of controls and patients applying the threshold-free cluster enhancement (TFCE) method (41, 49). Voxels from spatially co-registered binary lesion masks were excluded from the TBSS analyses. Design matrices included age and gender as covariates, and statistical significance was set at *p* < 0.0125 FWE to correct for testing for four diffusivity parameters.

In addition to the above quantitative analyses and whole-brain lesion load estimation, infratentorial lesion patterns were also evaluated visually based on FLAIR hyperintensities and the percentage of patients with lesions in the mesencephalon, pons, medulla, cerebellar peduncles, cerebellar hemispheres were documented.

## Results

### Gray matter analyses

**Figure 1:**
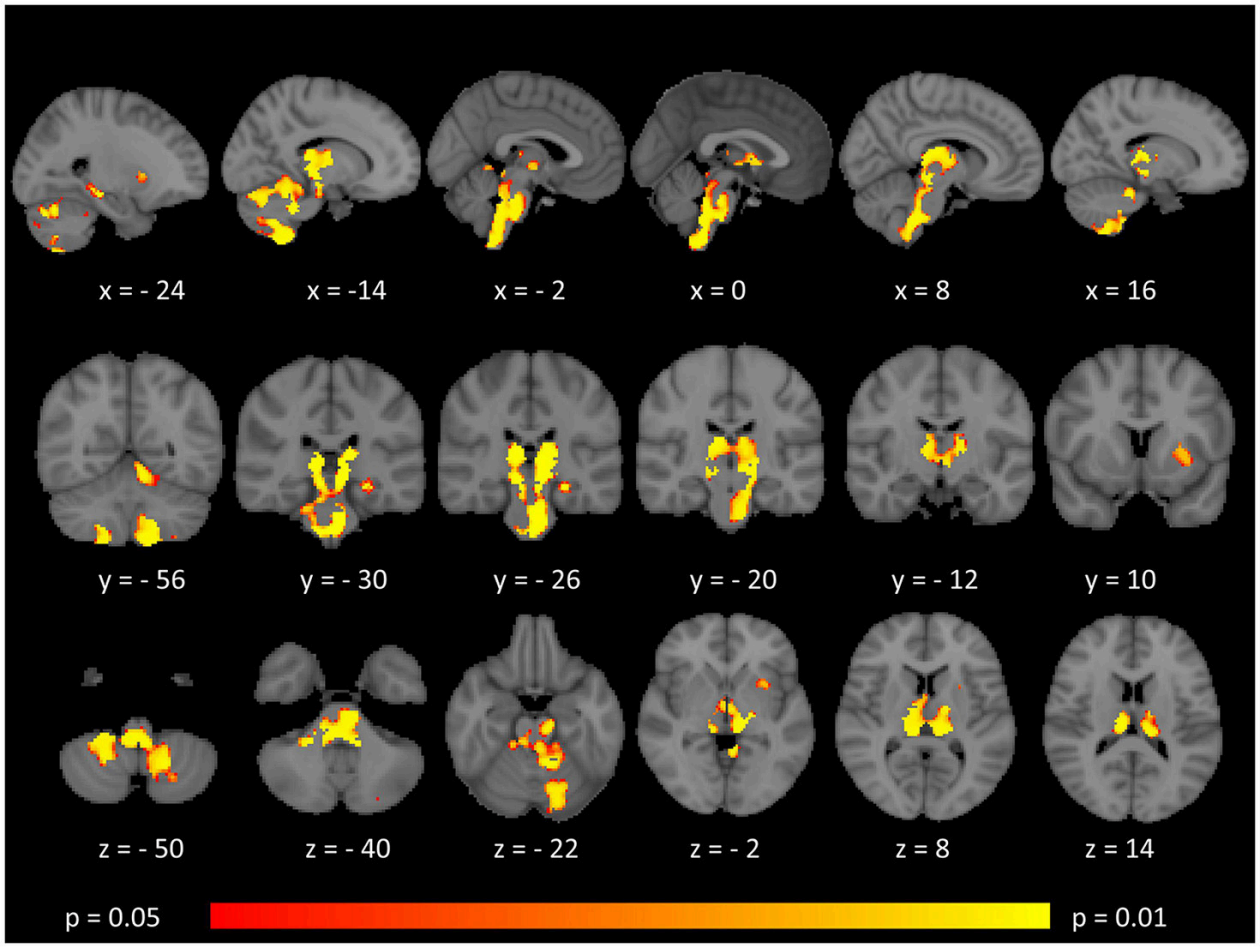
Patterns of gray matter atrophy in multiple sclerosis patients with gave-evoked nystagmus compared to healthy controls identified by voxel-based morphometry at *p* < 0.05 FWE adjusted for age and gender. Representative axial, coronal, and sagittal views are presented with corresponding MNI coordinates.

While surface-based morphometry did not identify statistically significant cortical thickness alterations in our MS cohort, voxel-based morphometry highlighted a pattern of gray matter degeneration involving bilateral cerebellar, brainstem, and medial thalamic regions. Additional subcortical gray matter pathology was identified in the left putamen and left hippocampus. At *p* < 0.05 FWE, no frontal, parietal, or temporal cortical changes have been captured (Figure 1).

### White matter analyses

The mean lesion load volume was 0.86 ± 0.34% of brain tissue volume (9.78 ± 4.07 mL) in the 31 MS patients who had structural data and 0.81 ± 0.32% of brain tissue volume (9.31 ± 4.04 mL) in the 22 MS patients who had diffusion tensor imaging data.

**Figure 2:**
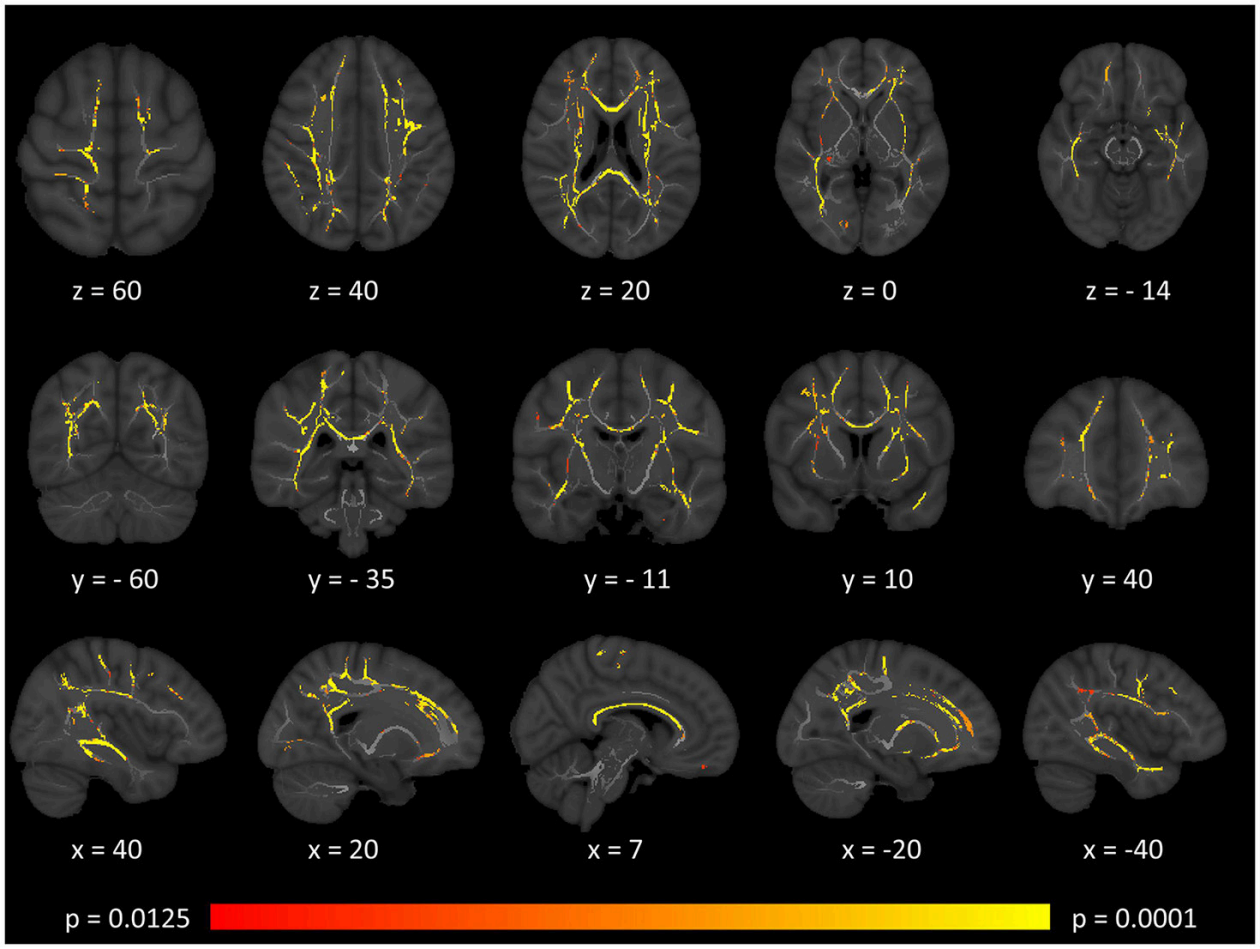
Patterns of increased axial diffusivity (AD) in multiple sclerosis patients with gave-evoked nystagmus compared to healthy controls at *p* < 0.0125 FWE adjusted for age and gender following tract-based spatial statistics. Representative axial, coronal, and sagittal views are presented with corresponding MNI coordinates.

**Figure 3:**
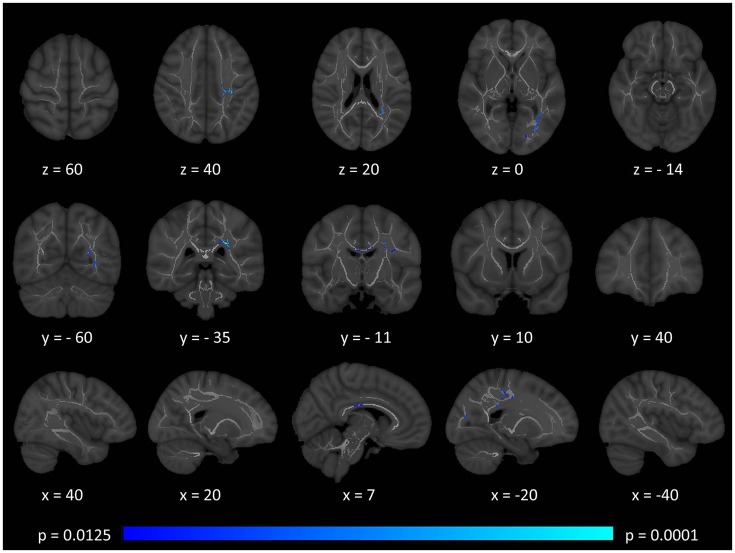
Patterns of decreased fractional anisotropy (FA) in multiple sclerosis patients with gave-evoked nystagmus compared to healthy controls at *p* < 0.0125 FWE adjusted for age and gender following tract-based spatial statistics. Representative axial, coronal, and sagittal views are presented with corresponding MNI coordinates.

**Figure 4:**
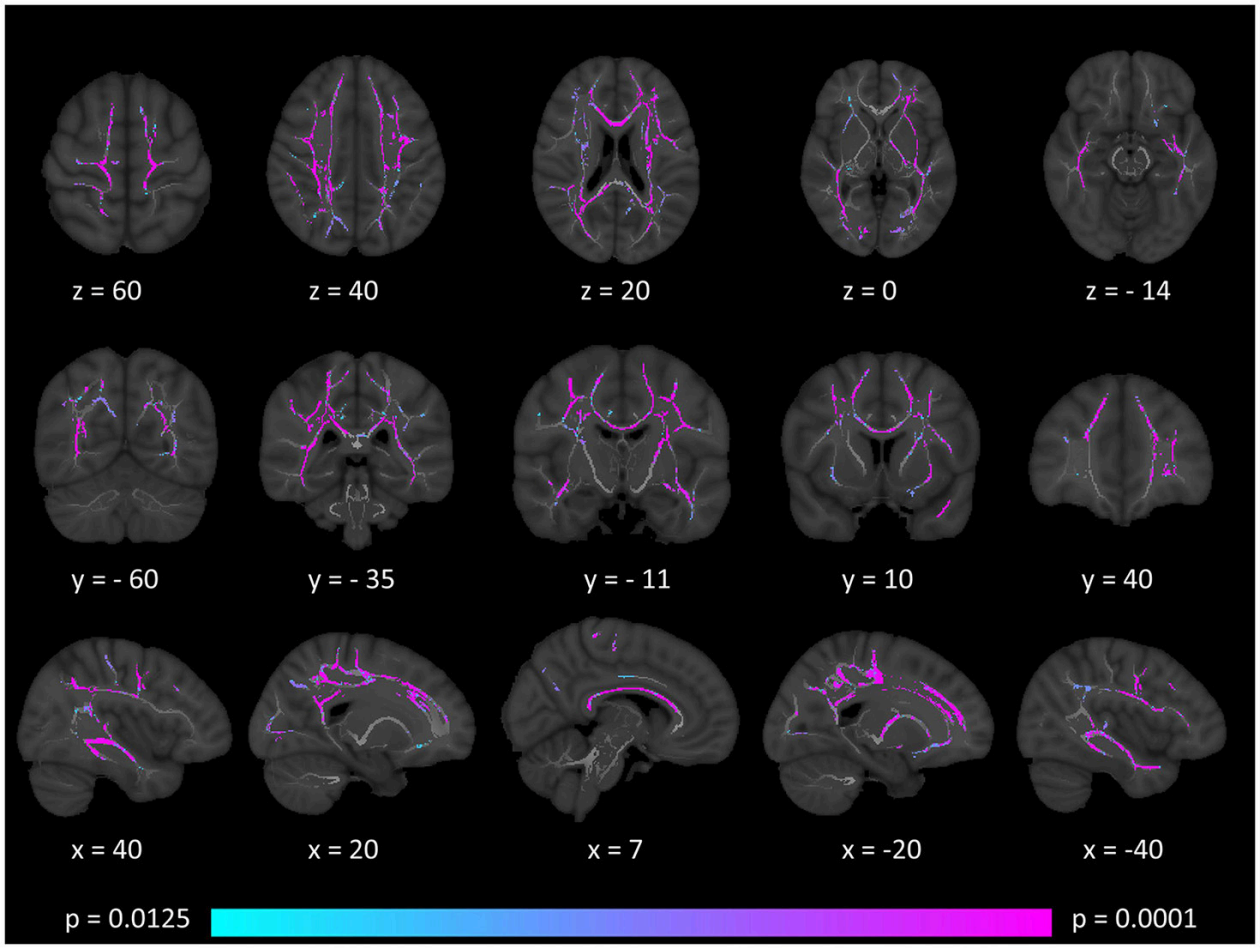
Patterns of increased mean diffusivity (MD) in multiple sclerosis patients with gave-evoked nystagmus compared to healthy controls at *p* < 0.0125 FWE adjusted for age and gender following tract-based spatial statistics. Representative axial, coronal, and sagittal views are presented with corresponding MNI coordinates.

**Figure 5:**
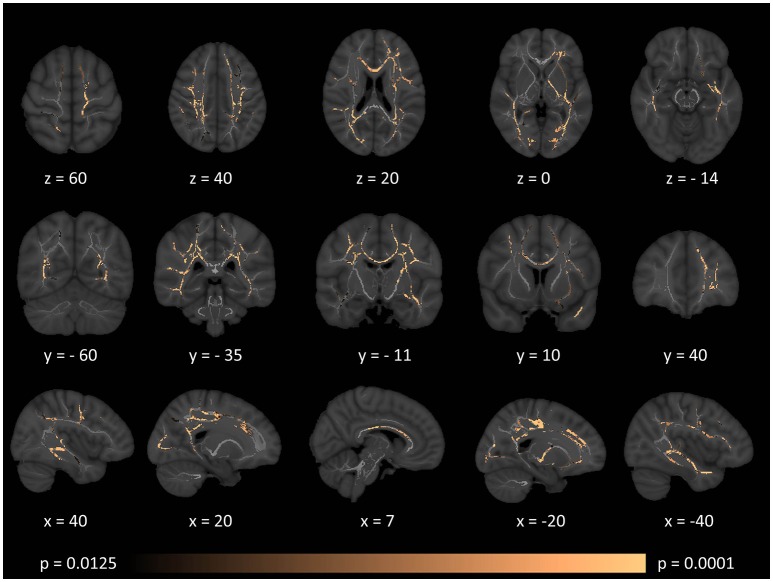
Patterns of increased radial diffusivity (RD) in multiple sclerosis patients with gave-evoked nystagmus compared to healthy controls at *p* < 0.0125 FWE adjusted for age and gender following tract-based spatial statistics. Representative axial, coronal, and sagittal views are presented with corresponding MNI coordinates.

Tract-based spatial statistics confirmed widespread multi-lobar white matter degeneration for all four diffusivity metrics at *p* < 0.0125 FWE, defining the statistical threshold based on Bonferroni adjustments (Figures 2–**5**). The most widespread diffusivity changes were identified by axial diffusivity (AD) analyses (Figure 2). Statistical maps of fractional anisotropy (FA) only identified relatively focal changes confined to the body of the corpus callosum, left superior corticospinal tract, and the occipital lobe at this threshold (Figure 3). Maps of mean diffusivity (MD) highlighted symmetric frontal, parietal, temporal, and occipital white matter alterations (Figure 4) which were more widespread than those observed on radial diffusivity (RD) maps (Figure 5). Interestingly, none of the four diffusivity metrics captured infratentorial white matter changes in the brain stem or cerebellum at the above statistical thresholds.

FLAIR hyperintensities were detected in the mesencephalon in 22.5% of the patients, in the pons in 25.8% of the patients, in the medulla in 19.3% of the patients, in the cerebellar peduncles in 29% of the patients, in the cerebellar hemispheres in 35.4% of the patients. No obvious infratentorial FLAIR hyperintensities were visible in 54.8% of the patients.

### Subcortical gray matter volumes

**Figure 6:**
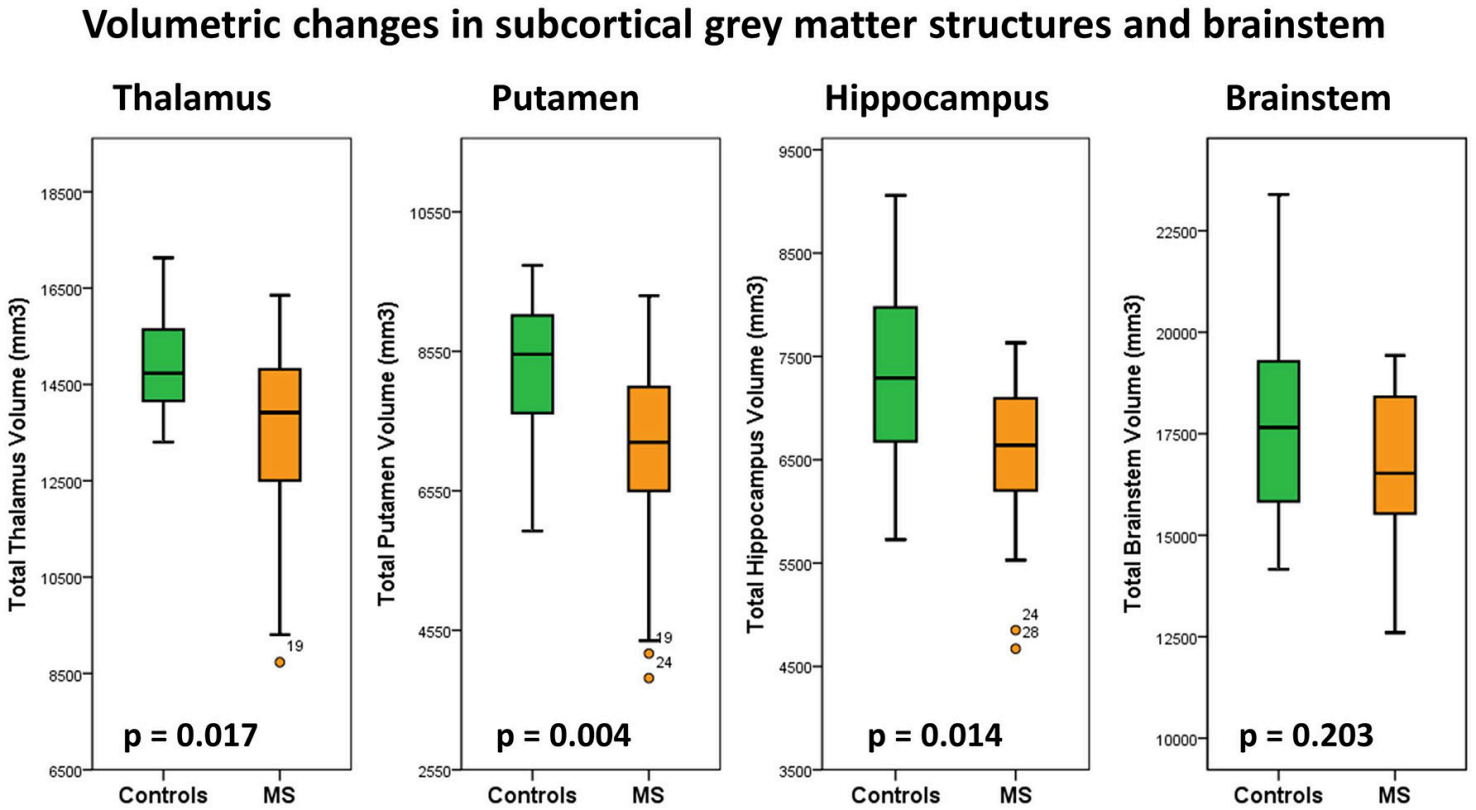
Total thalamus, putamen, hippocampus and brain stem volumes in healthy controls (green) and multiple sclerosis patients with gaze-evoked nystagmus (orange). *P*-values are corrected for age, gender and total intracranial volume. A statistical threshold of *p* < 0.0125 is considered significant following Bonferroni corrections for multiple testing.

Subcortical structures highlighted by voxel-based morphometry were further evaluated by volume estimations. *Post-hoc* comparisons of subcortical gray matter volumes (ANCOVA) highlighted significant putamen volume reductions in MS patients compared to controls. Considering the Bonferroni corrected threshold of *p* < 0.0125 a trend of thalamic (*p* = 0.017) and hippocampal (*p* = 0.014) atrophy was also identified. Table 2. The volumetric profiles of the four subcortical gray matter regions are further illustrated by box plots in Figure 6.

**Table 2:** The volumetric profile of subcortical gray matter structures and the brainstem in MS patients and controls.

	**Healthy Controls**		**Multiple Sclerosis**		***p*-value**	**partial η2**
	**Est. marg. mean**	**Std. error**	**Est. marg. mean**	**Std. error**		
Thalamus (mm^3^)	14954.89	432.196	13529.48	315.558	0.017	0.123
Putamen (mm^3^)	8571.63	368.478	7072.19	269.036	0.004	0.176
Hippocampus (mm^3^)	7326.01	234.217	6534.98	171.008	0.014	0.128
Brainstem (mm^3^)	17409.65	345.504	16817.92	252.262	0.203	0.036

*Estimated marginal means, standard error, comparative p-values and effect sizes are shown following corrections for age, gender and total intracranial volume. The statistical threshold was set at p < 0.0125 following Bonferroni corrections for multiple testing*.

## Discussion

Our findings highlight extensive cerebellar, brainstem and subcortical gray matter degeneration in a cohort of MS patients with gaze-evoked nystagmus. We also show extensive supratentorial white matter degeneration using four diffusivity measures. Cerebellar and brain stem degeneration in our cohort is dominated by gray matter pathology which was readily identified by standard, whole-brain analysis pipelines. Our supplementary volumetric analyses have captured thalamic, putaminal, and hippocampal pathology.

### Gray matter findings

Our standard morphometry analyses revealed bi-thalamic, bi-cerebellar and brainstem density reductions consistent with established nystagmus-associated anatomical foci. Additional gray matter degeneration was identified in the left putamen and left hippocampus. “Whole-brain” morphometry did not identify additional cortical pathology in either hemisphere. Similarly, surface-based morphometry did not identify cortical thickness alterations over the cerebral convexities.

Brainstem density reductions in the mesencephalon included the superior colliculus, a key structure in horizontal saccade control. The pontine changes incorporated the paramedian pontine reticular formation. The posterior medullary changes are consistent with the location of the nucleus prepositus hypoglossi and vestibular nuclei including the medial vestibular nucleus. The lack of significant cortical pathology suggests that the superior saccade pathway in our cohort is relatively intact and that the observed clinical signs are primarily driven by neural integrator network pathology in the brainstem (23, 50). The extensive brainstem gray matter pathology detected by VBM was not associated with volume reductions, (Table 2) tract-wise diffusivity alterations, (Figures 2–5) or widespread infratentorial lesion load on FLAIR.

Our gray matter analyses also map nystagmus-associated foci to medial cerebellar gray matter structures with relative sparing of the lateral and posterior aspects of the cerebellum. Pathology of specific cerebellar regions has been associated with specific types of nystagmus. Pathology of the flocculus and paraflocculus is traditionally linked to impaired suppression of the horizontal vestibulo-ocular reflex (VOR) during combined eye-head tracking (51). Nodulus and uvula pathology can result in positional nystagmus, periodic alternating nystagmus, or downbeat nystagmus. Gaze-evoked nystagmus is widely regarded as a manifestation of neural integrator network dysfunction (52, 53). In saccadic control a “pulse” and a “step” command is typically distinguished for neuronal discharge (54). The nucleus prepositus hypoglossi and to a lesser extent the medial vestibular nucleus contribute the “step” phase of neuronal activity. The superior colliculus in the midbrain controls saccade generation through pontine and midbrain pulse–step generator circuits and receives feedback from the brainstem burst generators (54). The role of midline cerebellar circuits is also well established in providing feed-back to burst generation (54). The etiology of GEN is linked to the inadequate “step” component, resulting in the eyes drifting back to their primary position and triggering a corrective saccade (22).

### White matter findings

Tract-based spatial statistics (TBSS) demonstrated extensive bilateral supratentorial white matter degeneration in our cohort of patients. The divergent patterns of FA, AD, MD, and RD alterations highlight the value of multiparametric diffusion tensor imaging in contrast to relying on a single diffusivity metric. In our cohort, FA analyses highlighted anatomically limited white matter degeneration compared to the more extensive patterns identified by AD, MD, and RD. FA and MD are composite proxies of white matter integrity defined based on the three eigenvalues. While axial diffusivity is often regarded as an axonal marker, (55, 56) and radial diffusivity as a myelin related measure, (57, 58) this may be a simplistic interpretation especially in the absence of spatially matched histopathological data (59). The identification of the principle eigenvector and the accurate estimation of the three eigenvalues are particularly challenging in crossing fibers, low signal-to-noise sequences and in the presence of focal pathology (60, 61). Therefore, while AD and RD adds to the characterisation of GEN-associated white matter changes, the categorical interpretation of AD and RD alterations as axonal- vs. myelin-related pathology is not justified.

Our standard, “whole-brain” TBSS analyses have not captured cerebellar or brainstem white matter changes (Figures 2–5). Given the sensitivity of multiparametric (AD, MD, RD, FA) white matter imaging to detect white matter alterations, this may suggest that in our cohort of patients, infratentorial pathology is dominated by gray matter degeneration. (Figure 1) The qualitative appraisal of lesion patterns also revealed that 54.8% percent of our patients had no visible infratentorial FLAIR hyperintensities and only 22.5% of the patients had mesencephalic and 25.8% pontine lesions. The discrepancy between the limited infratentorial pathology seen on visual inspection and the considerable gray matter degeneration detected by VBM highlights the limitation of qualitative assessments. In our sample, we cannot attribute eye-movement abnormalities to gray matter pathology alone, as the infratentorial white matter lesions undoubtedly contribute to the clinical profile of our patients. The contribution of infratentorial white matter lesion patterns to specific eye-movement abnormalities has been studied across multiple conditions, including stroke, Arnold-Chiari malformations and multiple sclerosis (9, 62).

### Subcortical gray matter findings

We identified a trend of thalamic volume reduction in our MS cohort and VBM enabled the localisation of thalamic pathology to bilateral posterior-medial foci. There is accruing radiological, clinical and pathological evidence that thalamic changes can be detected early in the course of MS and may be associated with a range of clinical manifestations including fatigue, pain syndromes, cognitive, oculomotor, and motor disturbances (63–65). Whereas volumetric analyses only highlight global atrophy, our VBM analysis revealed focal medial thalamus, posterior hippocampus and lateral putamen pathology.

The involvement of putamen and hippocampus illustrates the spectrum of extra-motor degeneration in MS and signals two candidate regions-of-interest which could be specifically explored in longitudinal studies and clinical trials. Hippocampal atrophy has been previously linked to cornu ammonis degeneration and associated with learning and memory encoding difficulties (11, 12, 66). Putamen pathology is relatively rarely reported in imaging studies of MS (67) and extrapyramidal symptoms may be particularly difficult to ascertain clinically in the presence of extensive upper motor neuron signs. Despite extensive brainstem density alterations on VBM, no brainstem volume reductions were detected by our segmentation approach, which highlights the benefit of using multiple complementary imaging modalities.

### Implications for clinical applications and pharmacological trials

In a clinical setting, scans of a given patient are often only qualitatively reviewed and lesion load is mostly visually estimated. The addition of 3D structural sequences without slice gaps and diffusion tensor sequences to clinical protocols may enable quantitative assessments and the accurate measurement of longitudinal change in the same patient. Magnetic resonance imaging is a key outcome measure in pharmaceutical trials, (68) and lesion load estimation based on T2-weighted imaging traditionally serves as a secondary endpoint for phase III clinical trials (69). In accordance with consensus guidelines, (70) recent clinical trials increasingly include cortical gray matter measures (71, 72). With very few exceptions however, (73) gray matter metrics of subcortical gray matter structures and the cerebellum are seldom utilized in clinical trials. The considerable cerebellar and deep gray matter degeneration highlighted by our study suggest that measures of these regions should also be evaluated as candidate biomarkers in MS. Cerebellar pathology is often exclusively linked to deficits in coordination and nystagmus, but its role in pseudobulbar affect and a range of cognitive functions is increasingly acknowledged (74, 75). An additional benefit of using quantitative metrics stems from their potential use in deep-learning and machine-learning applications which rely on advanced pattern recognition algorithms to aid diagnosis, patient stratification, and prognostication (76).

### Limitations

The main limitations of this proof-of-concept study lie in its small cohort size and the lack of eye movement recordings which would have allowed more detailed eye movement analyses and clinico-radiological correlations. Despite the stringent recruitment criteria, our patients inherently exhibit a degree of clinical heterogeneity as evidenced by their EDSS and disease-duration profile. While we cannot claim clinical homogeneity in our sample, the “common denominator” from a symptoms-perspective is the presence of gaze-evoked nystagmus. Accordingly, an important expansion of this study would be the inclusion of an MS cohort without nystagmus as a “disease-control” group to confirm the specificity of our infratentorial findings as a GEN-associated neuroimaging signature. Another obvious expansion of this study would be the longitudinal follow-up of the study participants to characterize patterns of progressive gray matter degeneration, response to therapy and rate of decline.

## Conclusions

Gaze-evoked nystagmus in multiple sclerosis is associated with multifocal gray matter pathology involving cerebellar, subcortical, and brainstem regions. White matter alterations in MS patients with gaze-evoked nystagmus are not limited to commissural tracts but involve widespread bi-hemispheric supratentorial regions. The imaging signature of gaze-evoked nystagmus in MS includes key structures of the oculomotor neural integrator network.

## Ethics statement

All procedures were performed in accordance with the ethical standards of the institutional ethics committee (SJH/AMNCH – St James's Hospital, Dublin, Ireland) and with the 1964 Helsinki declaration and its later amendments.

## Author contributions

PB, EF, RC, SLHS: study design, drafting the manuscript, data processing, statistical analyses, generation of figures and tables; JL: data processing, recruitment of participants, revision of the manuscript; JM: conceptualization and design of the study, data interpretation, revision of the manuscript; JR: conceptualization and design of the study, drafting and revision of the manuscript, supervision of all clinical aspects of the study, principle investigator.

### Conflict of interest statement

The authors declare that the research was conducted in the absence of any commercial or financial relationships that could be construed as a potential conflict of interest.

## Abbreviations

AD: Axial diffusivity
BG: Basal ganglia
CST: Corticospinal tract
DTI: Diffusion tensor imaging
EBN: Excitatory burst neurons
EDSS: Expanded Disability Status Scale
EPI: Echo-planar imaging
EPN: End-point nystagmus
FA: Fractional anisotropy
FAST: FMRIB's Automated Segmentation Tool
FDR: False discovery rate
FLAIR: Fluid-attenuated inversion recovery
FLIRT: FMRIB's Linear Image Registration Tool
FOV: Field-of-view
FEW: Familywise error
GEN: Gaze-evoked nystagmus
GM: gray matter
HC: Healthy control
MD: mean diffusivity
MLF: Medial longitudinal fasciculus
MNI152: Montreal Neurological Institute standard space
MS: Multiple sclerosis
MVN: Medial vestibular nucleus
NIN: Neural integrator network
NPH: Nucleus prepositus hypoglossi
PPMS: Primary progressive MS
PPRF: Paramedian pontine reticular formation
RD: Radial diffusivity
ROI: Region-of-interest
RRMS: Relapsing-remitting MS
SBM: Surface-based morphometry
SC: Superior colliculus
SPMS: Secondary progressive MS
TBSS: Tract-based spatial statistics
TE: Echo time
TFCE: Threshold-free cluster enhancement
TI: Inversion time
TR: Repetition time
T1WI: T1-weighted imaging
VBM: Voxel-based morphometry
WM: White matter.
